# The Correlation between Microsatellite Instability and the Features of Sporadic Colorectal Cancer in the North Part of Iran

**DOI:** 10.1155/2012/756263

**Published:** 2012-11-19

**Authors:** Masoumeh Faghani, Saba Fakhrieh Asl, Fariborz Mansour-Ghanaei, Keyvan Aminian, Alireza Tarang, Ramin Seighalani, Azadeh Javadi

**Affiliations:** ^1^Cellular and Molecular Research Center, Faculty of Medicine, Guilan University of Medical Sciences, Rasht, Iran; ^2^Gastrointestinal and Liver Diseases Research Center (GLDRC), Razi Hospital, Guilan University of Medical Sciences, Sardar-Jangle Avenue, P.O. Box 41448-95655, Rasht, Iran; ^3^Genomics & Animal Department, Agricultural Biotechnology Research Institute of North Region, Rasht, Iran

## Abstract

*Background*. The aim of this study was to determine the correlation between MSI and sporadic colorectal cancer in Guilan province, North part of Iran. *Materials and Methods*. A total of 96 patients who underwent resection for sporadic colorectal cancer in Guilan province were studied. No patients had positive family history of cancers. The frequencies of MSI were analyzed by testing the BAT-26 and BAT-25 markers. *Results*. MSI analysis revealed that 22.9% of the tumors (22 patients) were microsatellite instability positive and 77.1% (74 patients) were microsatellite instability negative. The highest rate of MSI (40.9%) was found in the rectal region. MSI-H status was seen more frequently in distal tumors (*P* = 0.04, odds ratio = 3.13, 0.96–10.14). *Conclusions*. Distal tumor location and MSI may associate with special clinicopathological features. It seems that there may be correlation with underlying genetic and immunologic mechanisms.

## 1. Introduction

Colorectal cancer (CRC) is one of the major public health problems in the world. CRC is one of the most common forms of cancer in western countries [1] and is the third most common cancer in males and the second in females [2]. Incidence of colorectal cancer in Iran's general population is between 7 and 8/100,000 in both men and women [3]. Incidence of CRC in Iran was lower than that of the western countries and is increasing in recent years [4].

Most of CRCs are sporadic, but 5% to 10% of colorectal cancers are associated with a primary genetic factor [5, 6]. Hereditary nonpolyposis colorectal cancer (HNPCC), the most common syndrome, is secondary to an inherited mutation in one of the DNA mismatch repair (MMR) genes (hMLH1, hMSH2, hMSH6, and hPMS2). Tumor classification is historically based on molecular features (e.g., microsatellite instability-high status (MSI-high) versus microsatellite stable (MSS)) [5, 6]. Failure of the DNA MMR system such as inactivation and mutation of MMR genes, mostly hMLH1 and hMSH2 that occur during the replication of DNA, causes MSI [7]. MSI is alteration in the length of simple, repetitive microsatellite sequences that occur throughout the whole genome [8].

MSI is detected in about 15% of all colorectal cancers [7] three percent of these are associated with Lynch syndrome and the other 12% are related with CpG island methylator phenotype (CIMP). CRC with MSI has particular characteristics such as improved survival rates and better prognosis than MSS tumors [9]. They also have distinct sensitivity to the action of chemotherapy [10]. On the other hand, MSI tumors may interfere in cancer genesis pathways and this may cause similar clinical outcomes. There is evidence that MSI tumors have common features, and MSI cancers that arise in the proximal colon have frequently low differentiated in pathology diagnosis and in general have an improved prognosis compared with MSS colorectal cancers [11–13].

These strong biological associations make MSI a useful genetic marker in patients with colorectal cancer. Therefore, we decided to evaluate the relationship between MSI and sporadic CRC in the northern part of Iran.

## 2. Patients and Methods

The sample included 96 specimens of sporadic colorectal adenocarcinoma and the corresponding adjacent cancer-free tissues from the north part of Iran (Rasht). Cases were selected from the Department of Pathology between February 2007 and December 2009 at the Razi and Poursina hospitals of Guilan University of Medical Sciences. Sporadic CRCs by definition are the patients who have no family history of cancer in first or second degree related families.

Prior to commencement of the study, all patients provided informed consent and the study was approved by our institutional ethics committee. Data collected from main charts and taking the history of patients included the presence or absence of lymph node metastases, tumor size and grade, patient age, and sex. The tumors classified “proximal” were from the cecum, hepatic flexure, ascending colon, and transverse colon, whereas “distal” tumors were from the splenic flexure, descending colon, sigmoid colon, and rectum.

MSI-H status was determined by PCR of genomic DNA that is isolated from paraffin-embedded normal and tumor tissues from each patient. Paraffin-tissue sections were deparaffinized in xylene then digested with proteinase K (2 mg/mL) overnight at 55°C. Using High Pure PCR Template Preparation DNA isolation kit (Roche, Germany), genomic DNA was extracted from samples, according to manufacturer's instructions. We used BAT-26 and BAT-25 markers because mononucleotide loci BAT-25 and BAT-26 have more than 90% sensitivity for detecting MSI by themselves; these markers are readily analyzed with routine staining methods as well as by PCR-SSCP [14, 15]. Between 50 to 100 nanograms of DNA was used as a template in a 25 *μ*L PCR reaction mixture containing 1.5 *μ*mol MgCl2, 1 U Taq polymerase (Sinagen, Iran), and 1 *μ*mol either of the primer pairs (Kawsar Biotech Company, Iran). For negative control no added DNA was included in each run. Forward and reverse primer sequences were used to amplify the BAT-26 and BAT-25 as follows [16, 17].

Bat-26: 5′-TGACTACTTTTGACTTCAGCC-3′ and 5′-AACCATTCAACATTTTTAACC-3′.

BAT-25: 5′-TCGCCTCCAAGAATGTAAGT-3′ and 5′-TCTGGATTTTAACTATGGCTC-3′.

PCR amplification was performed for these markers as follows: 1 cycle (94°C for 5 min), 35 cycles (30 sec at 94°C, 60 sec at 45°C for BAT-26 and 60 sec at 53°C for BAT-25, 60 sec at 72°C), 1 cycle (72°C for 5 min). The PCR product was denatured into single-strand DNA by heating in a formamide loading buffer (95% formamide, 10 mM EDTA, 0.05% bromophenol blue, 0.05% xylene cyanol) for 5 min at 94°C and then separating on a minigel electrophoresis apparatus (Farayand Danesh, Iran) using nondenaturing 15% acrylamide (60 : 1, acrylamide : bisacrylamide) gels containing 5% glycerol [16]. Gels were run at ambient temperature and without cooling. Gels were run for 2.5 h at 90 V. Silver staining was carried out as previously described. MSI-H was defined by the presence in the tumor samples of our allele compared to the adjacent normal tissues.

## 3. Statistical Analysis

The analyses were performed by the SPSS (version 16). Categorical variables (sex, site, histological grade, PTN, and metastasis of tumor) were compared with the *t*-test and Pearson chi-square test, and odds ratios (OR) were adjusted with 95% confidence intervals (95% CI). Continuous variables (age of recognized and size of tumor) were compared with the one-way ANOVA test. The Kolmogorov-Smirnov test (K-S test) was used for the equality of continuous variables. All *P* values were two tailed and the statistical significance level was set to *P* < 0.05.

## 4. Results

This analysis included 96 sporadic colorectal adenocarcinomas and adjacent normal colorectal tissue. The age of our patients (54 males, 42 females) ranged from 27 to 85 years (mean 63.5).

The main clinicopathological features of the patients are summarized in Tables 1 and 2. The mean age was 59.23 ± 16.82 years in MSI positive patients and 61.8 ± 13.4 years in MSI negative ones. There was not a statistical correlation between MSI and the age of the patients (*P* = 0.098). Data analysis revealed 64.5% of sporadic CRCs samples located in distal or left colic (Table 3). The distribution of lymph node metastasis in distal CRCs was not statistically different with right CRC patients (*P* > 0.05) (Table 2).

Analyze of all tumor tissue with MSI-H exhibited that all of these had a shortening of the poly (T) tract at BAT-25 and poly (A) tract at BAT-26. Data showed that all of the samples were heterozygous for these markers, which mean one allele within the normal range of repeat length. Silver stain PCR-SSCP analysis showed a specific PCR product of 124 and 121 bp for BAT-25 and BAT-26, respectively. Those cases with an unequivocally distinct additional band or shift in the tumor tissue DNA compared with normal tissue. MSI analysis revealed that 22 patients (22.9%) were MSI positive and 74 patients (77.1%) were MSI negative. The allelic frequencies for BAT-25 and BAT-26 were almost equivalent in our sample. Among our patients, 21 (21.9%) had alteration in BAT-26 marker, 20 (20.8%) had alteration in BAT-25 marker, and 18 (18.7%) had both alterations. Data showed that most of the tumors with MSI-H were located in the left colic region and the highest rate of MSI-H (40.9%) was found in the rectum. The prevalence of MSI-H in other anatomical regions of large intestine is showed in Table 3. There was a correlation between MSI-H and the site of tumor (*P* = 0.04, Odds ratio = 3.06, 0.96–10.14). There was no relationship between MSI-H and the size of the tumor (Table 1). A significant correlation was observed between site and size of tumor (*P* = 0.004) (Table 2). In patients, 64.6% had lymph node metastases, and *χ* ^2^ test showed a significant relationship between the sex and lymph node metastases (*P* = 0.015, OR = 2.81, 1.18–6.73).

## 5. Discussion

Microsatellites are repeated sequences that are highly susceptible to misalignment during replication, resulting in a 100-fold increase in their mutation rate [18]. These noncoding mono- and dinucleotide repeats are scattered in the genome, accumulate alterations in MMR-deficient tumors, and are used to assess the MSI phenotype [19]. Repeated sequences, most often mononucleotide tracts, exist in a number of human genes, such as hMSH6 and hMSH2. In fact, a number of these genes have shown to be altered in MSI+ colorectal tumors. Some mutations like insertion or deletion in microsatellite repeats regions cause MSI and these phenomena resulted from failure of the MMR system that edits errors made during DNA replication [17]. Data showed that MMR components participate in the recognition of DNA damage caused by alkylating agents, platinum-based drugs including those used in cancer chemotherapy [19]. Generally the tumors are classified as MSI+ tumors if instability is observed at a proportion of microsatellite loci (30%), but some authors consider that testing BAT-26 is almost adequate for MSI+ status [17]. So, detection of MSI, a functional marker of MMR defects, might be useful for determination of these defects [16].

On the other hand, there is evidence that MSI+ tumors have some significant biological and clinical importance [20, 21]. Some researchers believed that recognition of MSI can help us to understand the pathogenesis of cancer and is a useful thing in planning for prevention of cancer. Others showed that MSI should be important factor and may also be predictive of tumor responsiveness to certain chemotherapeutic drugs, such as alkylating agents and platinum-based drugs [22–26]. There are several studies in the literature investigating MSI in colorectal and other cancers at different loci by using different microsatellite markers [26]. In this study we used BAT-25 and BAT-26 markers to detect MSI-H tumors and we find that the ratios of these two markers are very similar to each other. We used silver staining method for detecting of MSI-H and it has been shown that silver stained SSCP gels can readily detect deletions of as little as 3 bp in BAT-26 [17]. This result was consistent with the previous study from Kim et al. [25], which reported that 67.3% of distal sporadic MSI-high CRCs and 94.2% of proximal MSI-high CRCs showed BAT-26 variation and 67.3% of distal sporadic MSI-high CRCs and 93% of proximal MSI-H CRCs showed BAT-25 variation. Our study result also revealed that 81.8% of total MSI-H had distal CRCs (Table 1). The distribution of Lymph node metastasis (LNM) was different in two groups (25.8% of proximal MSI-H CRCs and 74.2% of distal MSI-H CRCs had LNM).

Based on articles the rate of MSI in sporadic CRCs was 15–20% and these patients have deficiencies in DNA mismatch repair due to methylation silencing of the MLH1 gene [26, 27]. Recently, it has been shown that overexpression of hMSH2 or hMLH1 can induce apoptosis [28]. This may cause MSI+ sporadic CRCs to lose the ability to undergo efficient apoptosis. A total of 22.9% of the tumors in our study had MSI-H and as we pointed before all patients had no obvious family history. This is very different from results of similar studies in other countries such as Japan; Ichikawa et al. described that DNA variants of BAT-25 locus is very low in the Japanese population (as low as than 1%), but in African American people data showed that approximately 18.4% of African-Americans would be polymorphic at BAT-25 and 12.6% at the poly (A) tract at BAT-26 in 12.6% [29, 30]. This may show an ethnic difference in frequency of BAT-25 locus; in our study, a total of 20. 8% of CRCs had alteration in the size of BAT-25 marker.

In a previous study in Iran data showed that familiar clustering of colorectal cancer was located in the right side of the colon [31]. In our study most of sporadic CRCs samples were located in the left side of the colon (64.6%). In addition, the results of our study demonstrate that left colic MSI-H tumors must need a special attention than right MSI-H tumors. The first possible explanation for our results may by that 90.8% of left CRCs have pt3 and pt4 stages in pathological determination. Secondly in both right and left CRCs the degree of metastasis in MSI-H was higher than in MSS tumors (62.2 and 72.7% in right and left colic, resp.). However, in another study in Norway, one researcher demonstrated that proximal location of the tumor and MSI is related to a higher number of lymph nodes harvested [32]. A total of 64.58% of the patients in our study had lymph node metastasis which is very high compared to data from other countries at the time of the resection of tumor [32–35]. This difference may be due to a late diagnosis of cancer in Iran or the small sample size of our study. In contrast with Ansari et. al. article, most of our patients (80.2%) were above 50 years old [36].

In conclusion the results of this study indicate that left colic sporadic MSI-H tumors may have special clinicopathological features. Therefore recognition of MSI can help understand the pathogenesis and potentially plan screening and prevention of colorectal cancer. Future larger sample size population-based studies in other provinces of Iran are required to confirm the results of this study in Iranian people.

## Figures and Tables

**Table 1:** Clinicopathological features of colorectal cancers patients according to microsatellite instability status.

	MSS CRC	MSI CRC	*P* value
	(*n* = 74)	(*n* = 22)	(MSI versus MSS)
Age			0.09
Mean ± SD	61.88 ± 13.4	59.23 ± 16.8	
Median (range)	63.5 (27–85)	62 (31–85)	
Gender (%)			0.29
Male	40 (54.1)	14 (63.6)	
Female	34 (45.9)	8 (36.4)	
Tumor location (%)			0.04*
Proximal	30 (40.5)	4 (18.2)	
Distal	44 (59.5)	18 (81.8)	
Metastasis of lymph node (%)			0.259
Yes	46 (62.2)	16 (72.7)	
No	28 (37.8)	6 (27.3)	
Tumor size (%)			0.595
<20 mm	45 (60.8)	11 (50.0)	
20–50 mm	23 (31.1)	8 (36.4)	
>50 mm	6 (8.1)	3 (13.6)	
Tumor grade (%)			0.418
Well differentiated	35 ( 47.3)	13 (59.1)	
Moderate differentiated	37 (50.0)	7 (31.8)	
Poor differentiated	2 (2.7)	2 (9.1)	

MSI: microsatellite instability, MSS: microsatellite stable, CRC: colorectal cancer, SD: standard deviation, *n*: number.

*Significant.

**Table 2:** Clinicopathological features of colorectal cancers patients according to site of tumor.

	Left CRC	Right CRC	*P* value
	(*n* = 66)	(*n* = 30)	(left versus right)
Age			0.19
Mean ± SD	62.58 ± 13.86	58.4 ± 14.7	
Median (range)	64.5 (27–85)	60 (27–85)	
Gender (%)			0.43
Male	38 (57.6)	16 (53.3)	
Female	28 (42.4)	14 (46.7)	
Metastasis of lymph node (%)			0.259
Yes	46 (62.2)	16 (72.7)	
No	28 (37.8)	6 (27.3)	
Tumor size (%)			0.004*
<20 mm	44 (66.7)	12 (40.0)	
20–50 mm	19 (28.8)	12 (40.0)	
>50 mm	3 (4.5)	6 (20.0)	

CRC: colorectal cancer, SD: standard deviation, *n*: number.

*Significant.

**Table 3:** The prevalence of MSI in the anatomical regions of large intestine.

MSI	Site of tumor								Total
	Secum *n* (%)	Ascending colon *n* (%)	Right colic flexure *n* (%)	Transverse *n* (%)	Left colic flexure *n* (%)	Descending colon *n* (%)	Sigmoid *n* (%)	Rectum *n* (%)	
Positive	4 (18.2)	0	1 (4.5)	1 (4.5)	1 (4.5)	3 (13.6)	3 (13.6)	9 (40.9)	22
Negative	10 (13.5)	11 (14.9)	4 (5.4)	3 (4.1)	2 (2.7)	5 (6.8)	22 (29.7)	17 (23.0)	74

## References

[1] Parkin DM, Bray F, Ferlay J, Pisani P (2005). Global cancer statistics, 2002. *CA Cancer Journal for Clinicians*.

[2] Jemal A, Bray F, Center MM, Ferlay J, Ward E, Forman D (2011). Global cancer statistics. *CA Cancer Journal for Clinicians*.

[3] Mahdavinia M, Bishehsari F, Ansari R (2005). Family history of colorectal cancer in Iran. *BMC Cancer*.

[4] Kolahdoozan S, Sadjadi A, Radmard AR, Khademi H (2010). Five common cancers in Iran. *Archives of Iranian Medicine*.

[5] Redston M (2001). Carcinogenesis in the GI tract: from morphology to genetics and back again. *Modern Pathology*.

[6] Compton CC (2003). Colorectal carcinoma: diagnostic, prognostic, and molecular features. *Modern Pathology*.

[7] Thibodeau SN, Bren G, Schaid D (1993). Microsatellite instability in cancer of the proximal colon. *Science*.

[8] Boland CR, Thibodeau SN, Hamilton SR (1998). A National Cancer Institute workshop on microsatellite instability for cancer detection and familial predisposition: development of international criteria for the determination of microsatellite instability in colorectal cancer. *Cancer Research*.

[9] Popat S, Hubner R, Houlston RS (2005). Systematic review of microsatellite instability and colorectal cancer prognosis. *Journal of Clinical Oncology*.

[10] Watanabe T, Wu TT, Catalano PJ (2001). Molecular predictors of survival after adjuvant chemotherapy for colon cancer. *The New England Journal of Medicine*.

[11] Huang YQ, Yuan Y, Ge WT, Hu HG, Zhang SZ, Zheng S (2010). Comparative features of colorectal and gastric cancers with microsatellite instability in Chinese patients. *Journal of Zhejiang University B*.

[12] Sinicrope FA, Rego RL, Halling KC (2006). Prognostic impact of microsatellite instability and DNA ploidy in human colon carcinoma patients. *Gastroenterology*.

[13] Esemuede I, Forslund A, Khan SA (2010). Improved testing for microsatellite instability in colorectal cancer using a simplified 3-marker assay. *Annals of Surgical Oncology*.

[14] Redston M (2001). Carcinogenesis in the GI tract: from morphology to genetics and back again. *Modern Pathology*.

[15] Pan KF, Liu W, Lu YY (2003). High throughput detection of microsatellite instability by denaturing high-performance liquid chromatography. *Human Mutation*.

[16] Hatch SB, Lightfoot HM, Garwacki CP (2005). Microsatellite instability testing in colorectal carcinoma: choice of markers affects sensitivity of detection of mismatch repair-deficient tumors. *Clinical Cancer Research*.

[17] Nikbahkt Dastjerdi M, Faghani M, Salehi M, Rabbani M (2009). A preliminary study of microsatellite instability analysis in different genotypes of p53 codon 72 in breast invasive ductal carcinomas. *Medical Journal of the Islamic Republic of Iran*.

[18] Loukola A, Eklin K, Laiho P (2001). Microsatellite marker analysis in screening for hereditary nonpolyposis colorectal cancer (HNPCC). *Cancer Research*.

[19] Jacob S, Praz F (2002). DNA mismatch repair defects: role in colorectal carcinogenesis. *Biochimie*.

[20] Lage H, Dietel M (1999). Involvement of the DNA mismatch repair system in antineoplastic drug resistance. *Journal of Cancer Research and Clinical Oncology*.

[21] Azzoni C, Bottarelli L, Cecchini S, Silini EM, Bordi C, Sarli L (2011). Sporadic colorectal carcinomas with low-level microsatellite instability: a distinct subgroup with specific clinicopathological and molecular features. *International journal of colorectal disease*.

[22] Koi M, Umar A, Chauhan DP (1994). Human chromosome 3 corrects mismatch repair deficiency and microsatellite instability and reduces N-methyl-N’-nitro-N-nitrosoguanidine tolerance in colon tumor cells with homozygous hMLH1 mutation. *Cancer Research*.

[23] Fink D, Nebel S, Aebi S (1996). The role of DNA mismatch repair in platinum drug resistance. *Cancer Research*.

[24] Marra G, D’Atri S, Corti C (2001). Tolerance of human MSH2+/- lymphoblastoid cells to the methylating agent temozolomide. *Proceedings of the National Academy of Sciences of the United States of America*.

[25] Kim YH, Min BH, Kim SJ (2010). Difference between proximal and distal microsatellite-unstable sporadic colorectal cancers: analysis of clinicopathological and molecular features and prognoses. *Annals of Surgical Oncology*.

[26] Esemuede I, Forslund A, Khan SA (2010). Improved testing for microsatellite instability in colorectal cancer using a simplified 3-marker assay. *Annals of Surgical Oncology*.

[27] Wheeler JMD, Loukola A, Aaltonen LA, McC Mortensen NJ, Bodmer WF (2000). The role of hypermethylation of the hMLH1 promoter region in HNPCC versus MSI+ sporadic colorectal cancers. *Journal of Medical Genetics*.

[28] Zhang H, Richards B, Wilson T (1999). Apoptosis induced by overexpression of hMSH2 or hMLH1. *Cancer Research*.

[29] Ichikawa A, Sugano K, Fujita S (2001). DNA variants of BAT-25 in Japanese, a locus frequently used for analysis of microsatellite instability. *Japanese Journal of Clinical Oncology*.

[30] Pyatt R, Chadwick RB, Johnson CK, Adebamowo C, De La Chapelle A, Prior TW (1999). Polymorphic variation at the BAT-25 and BAT-26 loci in individuals of African origin: implications for microsatellite instability testing. *American Journal of Pathology*.

[31] Malekzadeh R, Bishehsari F, Mahdavinia M, Ansari R (2009). Epidemiology and molecular genetics of colorectal cancer in Iran: a review. *Archives of Iranian Medicine*.

[32] Søreide K, Nedrebø BS, Søreide JA, Slewa A, Kørner H (2009). Lymph node harvest in colon cancer: influence of microsatellite instability and proximal tumor location. *World Journal of Surgery*.

[33] Baxter NN, Virnig DJ, Rothenberger DA, Morris AM, Jessurun J, Virnig BA (2005). Lymph node evaluation in colorectal cancer patients: a population-based study. *Journal of the National Cancer Institute*.

[34] Tekkis PP, Smith JJ, Heriot AG, Darzi AW, Thompson MR, Stamatakis JD (2006). A national study on lymph node retrieval in resectional surgery for colorectal cancer. *Diseases of the Colon and Rectum*.

[35] Bui L, Rempel E, Reeson D, Simunovic M (2006). Lymph node counts, rates of positive lymph nodes, and patient survival for colon cancer surgery in Ontario, Canada: a population-based study. *Journal of Surgical Oncology*.

[36] Ansari R, Mahdavinia M, Sadjadi A (2006). Incidence and age distribution of colorectal cancer in Iran: results of a population-based cancer registry. *Cancer Letters*.

